# Expression of a tumor-associated gene, LASS2, in the human bladder carcinoma cell lines BIU-87, T24, EJ and EJ-M3

**DOI:** 10.3892/etm.2013.892

**Published:** 2013-01-11

**Authors:** QINGHUA ZHAO, HAIFENG WANG, MINGYING YANG, DELIN YANG, YIGANG ZUO, JIANSONG WANG

**Affiliations:** 1 Department of Gynaecology, The Second Affiliated Hospital of Kunming Medical University, Yunnan Institute of Urology, Kunming 650101, P.R. China; 2 Department of Urology, The Second Affiliated Hospital of Kunming Medical University, Yunnan Institute of Urology, Kunming 650101, P.R. China

**Keywords:** bladder cancer, *Homo sapiens* longevity assurance homolog 2 of yeast LAG1, expression, biological characteristics

## Abstract

*Homo sapiens* longevity assurance homolog 2 of yeast LAG1 (LASS2), a metastasis suppressor gene of human cancer, is the most abundantly expressed member of the ceramide synthase gene family. Expression of LASS2 has been reported in carcinomas of the prostate, liver and breast. However, there has been no report on the expression of LASS2 in human bladder cancer cell lines. In order to investigate the expression and potential role of this new tumor metastasis supressor gene in human bladder cancer, we compared the proliferation, metastasis and invasion among the BIU-87, T24, EJ and EJ-M3 human bladder cancer cell lines. The mRNA expression levels of the LASS2 gene were examined using real-time quantitative PCR (qPCR). The expression levels of LASS1 and LASS3 mRNA were used as references. The protein expression level of the LASS2 gene was detected using western blotting. The most aggressive of these four human cancer cell lines was observed to be EJ-M3. The expression of LASS2 mRNA was significantly correlated with diverse proliferation, metastasis and invasion. The expression levels of LASS1 and LASS3 mRNA were not correlated with these parameters. At the protein level, we observed that the more aggressive the cancer cell line, the lower the LASS2 protein expression level. Therefore, LASS2 expression may be correlated with the development and progression of human bladder cancer and may be a prognostic indicator for this cancer.

## Introduction

Bladder cancer is one category of common genitourinary cancers and recurrence with metastasis is the main reason for treatment failure (1). Metastases are the end result of tumor progression and the most common cause of mortality in cancer patients. The process of metastasis is multistep and interruption of this process at any individual step may arrest the metastatic cascade (2). Therefore, it is important to investigate the biological mechanisms contributing to the development and metastatic movement of bladder cancer.

Tumor metastasis suppressor genes are a relatively new class of genes. These genes, which include NM23 (3–7), KISS-1 (8–11) and RhoGDI2 (12–14), reduce the metastatic ability of cancer cells at orthotopic sites,. However, the mechanism of action and role in human cancer remains unknown for the majority of these genes.

The LASS (longevity assurance homolog) family members are highly conserved from yeasts to mammals. *Homo sapiens* longevity assurance homolog 2 of yeast LAG1 (LASS2), also known as tumor metastasis suppressor gene 1 (TMSG1, GenBank accession number AF189062), is a gene isolated from a human liver cDNA library by the laboratory of Shanghai Medical College, Fudan University (Shanghai, China), and it is a human homolog of the yeast (*Saccharomyces cerevisiae)* longevity assurance gene, LAG1. LASS2 has been observed to correlate with the degree of invasion and recurrence in carcinomas of the prostate (15,16), liver (17) and breast (18). However, there has been no report on the expression of LASS2 in human bladder cancer cell lines.

In our previous study, we demonstrated that LASS2-negative bladder cancer was associated with poor clinical prognosis. The expression of LASS2 mRNA was significantly correlated with clinical stage (P<0.001), depth of tumor invasion (P<0.001) and recurrence (P<0.001) (19).

In order to fully understand the biological importance of LASS2, we examined the mRNA and protein expression of LASS2 in human bladder cancer cell lines (BIU-87, T24, EJ and EJ-M3) with diverse proliferation and invasion potential, and analyzed the potential role of the tumor metastasis supressor gene LASS2 in these human bladder cancer cell lines.

## Materials and methods

### Cell lines and cell culture

The EJ, T24 and BIU-87 human bladder cancer cell lines were preserved by our department (Department of Urology, the Second Affiliated Hospital of Kunming Medical University, Yunnan Institute of Urology, China). The highly invasive human bladder carcinoma EJ-M3 cell line was established in previous studies (20,21). The BIU-87, T24, EJ and EJ-M3 cell lines were cultured in DMEM supplemented with 10% fetal bovine serum and incubated in 5% CO_2_/95% air at 37°C.

### Cell proliferation assay

The growth of the BIU-87, T24, EJ and EJ-m3 cells was evaluated by a cell proliferation assay. Briefly, cells (1×10^4^) were plated in seven 24-well culture plates after the cell count. The cells were harvested on days 1, 2, 3, 4, 5, 6 and 7 for cell counting experiments, and the values were normalized to untreated controls. This experiment was repeated three times. We then created growth curves and compared the proliferation ability among the four cell lines.

### Matrigel invasion assay

The assay was carried out according to the method of Girnita *et al*(22). The invasive ability of BIU-87, T24, EJ and EJ-M3 cells was evaluated using a Matrigel invasion assay. BD BioCoat Matrigel Invasion Chambers with 8-mm pore size PET membranes (BD Biosciences, San Jose, CA, USA) for 24-well plates were prepared by hydrating for 2 h at 37°C. A total of 2×10^5^ cells in 0.2 ml were seeded into each insert. After culturing for 12 h, the invasion chamber was removed and the medium in the top wells was aspirated and cells on the upper surface of the membranes were removed with cotton swabs. The invading cells which remained attached to the lower side of the membrane were removed by flushing with a pipette before migrating cells present in the bottom chambers were labeled. The fluorescence intensities were plotted on a standard histogram and the number of invading cells was calculated. All experiments were performed in triplicate. Data were expressed as the number of invaded cells.

### Total RNA isolation and real-time quantitative PCR (qPCR)

According to the manufacturer’s instructions, total RNA was extracted using TRIzol (Invitrogen, Carlsbad, CA, USA). First-strand cDNA was synthesized in a volume of 20 *μ*l using 1 *μ*g total RNA and TaqMan reverse transcription reagents (Applied Biosystems, Foster City, CA, USA). The target gene sequences were obtained from the National Center for Biotechnology Information GenBank databases. The purity of RNA samples was determined by the OD_260_/OD_280_ (between 1.7 and 2.0) and the OD_260_/OD_230_ (>1.7) values and by analysis of the ribosomal RNA band integrity by conventional denaturing agarose RNA electrophoresis (23). PCR was performed with an ABI PRISM 7000 (Applied Biosystems) and 2X qPCR MasterMix (Eurogentec, Seraing, Belgium) according to the manufacturer’s instructions. To quantify target mRNA levels, LASS1, LASS2 and LASS3 Genes Expression assays were purchased from Applied Biosystems. The primer sequences were as follows: LASS1 forward 5′-CACACACATCTTTCGGCCC-3′, and reverse 5′-ACCTGGCAGCATCTCTAGGC-3′; LASS2 forward 5′-TCTCCTGGTTTGCCAATTACG-3′, reverse 5′-CCGGGCAGGGACCCTCATCA-3′; LASS3 forward 5′- GAGCGCCAGGT TGA A AGATG-3′, and reverse 5′-GGAATTTCTGCAGCCTGCA-3′. All primers spanned an intron to ensure discrimination between cDNA and genomic DNA. The relative amount of specific mRNA was normalized to GAPDH using the following primer sequences: forward 5′-GGTCTCCTCTGACTTCAACA-3′, and reverse 5′-GAGGGTCTCTCTCTTCCT-3′. All PCR reactions were run in duplicate and were performed with 40 cycles. A dilution series was carried out for each gene and the 18S ribosomal subunit was used as an internal control. qPCR analysis was carried out using the 2^−^^ΔΔCt^ method (24).

### Western blot analysis

Western blots of whole-cell lysates from a known number of cells were prepared. The whole-cell lysates were made by lysing cells in the buffer. Protein (50 mg) was separated by SDS-PAGE gels and transferred to a membrane. LASS2 was detected in 100 *μ*g of protein from whole cell extracts, according to the procedure described by Baron *et al*(25) and Calogero *et al*(26), and actin was used as a loading control. The primary and secondary antibodies were purchased from Santa Cruz Biotechnology (Santa Cruz, CA, USA). The protein was visualized by enhanced chemoluminescence (ECL; Santa Cruz) and normalized with respect to the actin content of each sample.

### Statistical analysis

The results were expressed as mean ± the standard error of the mean (SEM) of at least three separate experiments. Statistical significance was assessed using a two-tailed unpaired Student’s t-test. The correlation between gene expression and potential causative variables, were evaluated with the Chi-square test. P<0.05 was considered to indicate a statistically significant result. Each statistical analysis was performed using the SPSS 11.0 software for Windows (SPSS Inc., Chicago, IL, USA).

## Results

### Growth curves

**Figure 1.**
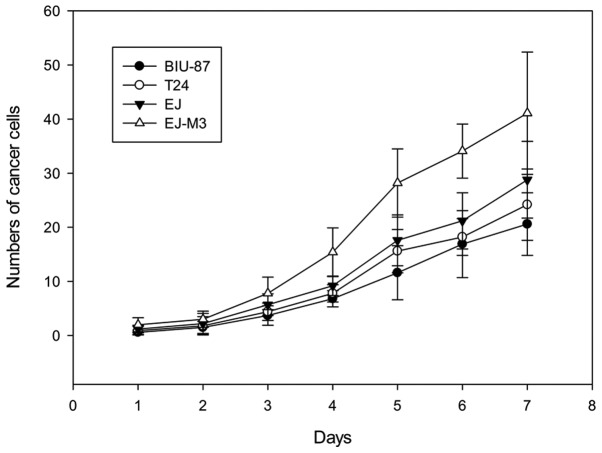
Growth curves of BIU-87, T24, EJ and EJ-M3 cells. The EJ-M3 cell proliferation was the fastest (P<0.001, vs. BIU-87, T24 and EJ) and BIU-87 cell proliferation was the slowest (P<0.05 vs. T24, EJ and EJ-M3).

The population-doubling time of BIU-87, EJ, T24 and EJ-M3 cell lines were 31.7±0.1, 27.6±0.2, 28.5h±0.1 and 20.8±0.2h, respectively. The cell growth rate was significantly different between EJ-M3 and the other three cell lines (P<0.05). There was no significant difference between the growth rates of BIU-87 and T24 (P>0.05). The growth curves are shown in Fig. 1.

### In vitro invasion

The numbers of EJ-M3, EJ, T24 and BIU-87 cells that attached to the lower side of the membrane were 122.8±16.8, 78.6±14.1, 32.9±10.7 and 16.0±8.1, respectively. The numbers of EJ-M3, EJ, T24 and BIU-87 cells that invaded into the 24-well plate were 32.8±9.8, 7.5±4.2, 10.2±5.1 and 8.0±4.9, respectively. Using the method of analysis of variance (ANOVA), we demonstrated that there was no statistic difference of invasiveness between EJ, T24 and BIU-87. However, the invasiveness of EJ-M3 was significantly different from that of EJ, T24 and BIU-87 (P<0.001) and are shown in Fig.s 2–4.

### qPCR analysis of LASS1, LASS2 and LASS3 mRNA expression in the EJ-M3, EJ, T24 and BIU-87 cell lines

**Figure 5.**
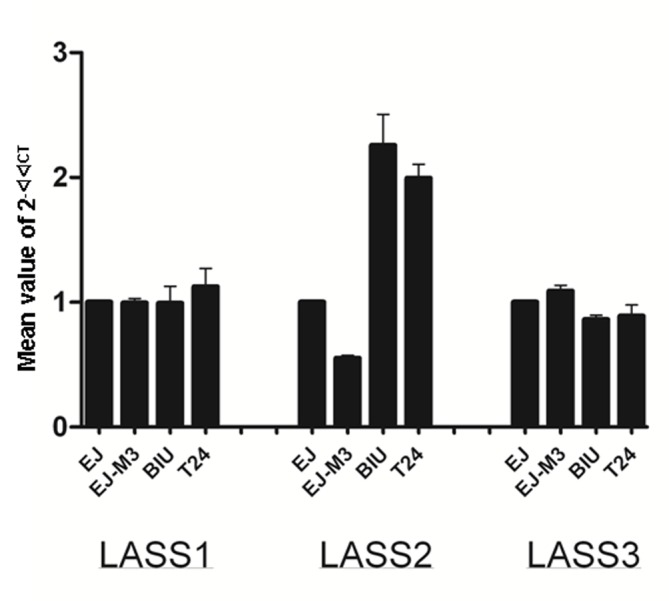
LASS1, LASS2 and LASS3 mRNA expression analysis. LASS1, LASS2 and LASS3 mRNA expression of BIU-87, EJ-M3, T24 and EJ cells was detected by quantitative real-time PCR.

Prior to the quantitative analysis, optimization procedures were carried out for qPCR reactions on EJ-M3, EJ, T24 and BIU-87 cell lines, using specific primers for human LASS2, LASS2 and LASS3 genes, separately. Since LASS1, LASS2 and LASS3 have been reported to be expressed at the mRNA level in normal human tissues, we used LASS2 expression as a control in qPCR reactions. To optimize our quantitative technique, a cDNA synthesized from the cell sample was used in serial dilution and a PCR efficiency close to 100% was obtained. After optimization of the qPCR, expression of LASS2, LASS2 and LASS3 genes were analyzed using qPCR in the bladder carcinoma cell lines EJ-M3, EJ, T24 and BIU-87. The result is shown in Fig. 5.

The differences among the four bladder carcinoma cell lines were calculated using the ANOVA method. The differences in LASS2 mRNA expression levels among the four cell lines were statistically significant (P<0.001). LASS1 and LASS3 expression levels among the four cell lines were not statistically significant (P>0.05).

### Western blot analysis of LASS2 protein expression in the EJ-M3, EJ, T24 and BIU-87 cell lines

**Figure 6.**
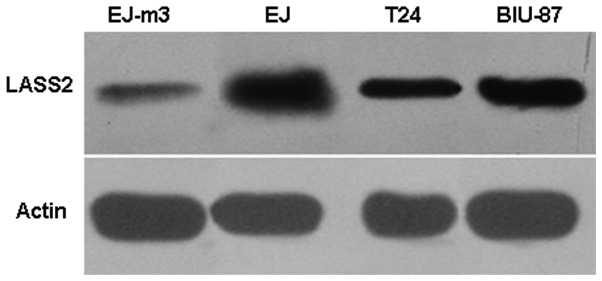
LASS2 protein expression analysis. LASS2 protein expression of BIU-87, EJ-M3, T24 and EJ cells was detected by western blotting. The difference in LASS2 protein expression among the four cell lines was statistically significant.

The expression of LASS2 protein was observed in the four bladder carcinoma cell lines, EJ-M3, EJ, T24 and BIU-87. The highest expression level of LASS2 was detected in the human bladder carcinoma cell line EJ-M3 by western blot analysis. Moderate LASS2 protein expression was observed in EJ and T24 cell lines, and weak LASS2 expression was observed in the BIU-87 cell line by western blotting. The result is shown in Fig. 6.

## Discussion

The present study identified the presence of LASS2 mRNA and protein in four bladder carcinoma cell lines. The expression levels of LASS2 among these human bladder cancer cell lines, which have diverse reproductive activity and invasive abilities, are distinct at the mRNA and protein level.

It is well known that an imbalance of cellular growth regulation results from certain genetic changes in the oncogenic process, which lead to uncontrolled tumor growth. However, unrestrained proliferation does not, by itself, result in invasion and metastasis. Certain additional genetic changes are required for tumor cells to be able to invade and metastasize (27). A tumor metastasis suppressor gene that is involved in tumor cell metastasis has been confirmed (28).

LASS2 has previously been demonstrated to function as a tumor metastasis suppressor gene. Previous studies have shown that LASS2 gene plays a key role in carcinomas of the prostate (15,16), liver (17) and breast (18).Chen *et al*(17) observed that transfection of LASS2 by lipofectamine inhibited the invasion and metastasis of a highly metastatic liver cancer cell line, HCCLM3. Su *et al*(15,16) demonstrated that overexpression of TMSG-1 (LASS2) inhibits the proliferation, anchorage-independent growth and invasion of a highly metastatic prostate cell line, PE-3M-1E8. We observed that LASS2-negative bladder cancer was associated with poor clinical prognosis and the expression of LASS2 was significantly correlated with clinical stage, depth of tumor invasion and recurrence in our preliminary studies (19).

This is the first study investigating the expression of LASS2 in human bladder carcinoma cell lines. In this study, we examined the protein and mRNA expression of LASS2 in four bladder carcinoma cell lines using the methods of qPCR and western blotting.

We also demonstrated that LASS2 expression correlated with the biological characteristics of these human bladder carcinoma cell lines. The results suggest that LASS2 expression is downregulated in cell lines with a high degree of malignancy and that the more malignant tumor cells expressed lower amounts of LASS2 at the protein and mRNA levels. This study supports the role of LASS2 as a metastasis suppressor gene and its potential utility as a clinical prognostic marker in human bladder carcinoma.

The results of qPCR are coincidental to the results of western blotting. LASS2 may be a useful indicator of tumor invasion and progression. However, due to the increasing appreciation of the importance of LASS2 in human bladder carcinoma, as well as other cancers, future studies focusing on how LASS2 is regulated and the mechanisms of its anti-metastatic functions, with the goal of critically examining the possibilities of exploiting LASS2 as a therapeutic target, are required.

In conclusion, using qPCR and western blotting, we have shown that LASS2 expression may be correlated with the development and progression of human bladder cancer and may be a prognostic indicator for this cancer.

## Figures and Tables

**Figure 2.**
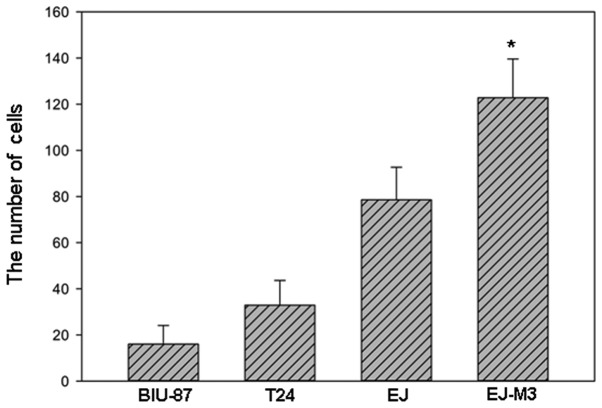
Numbers of EJ-M3, EJ, T24 and BIU-87 cells that attached to the lower side of the membrane. (^*^P<0.05, vs BIU-87, T24 and EJ).

**Figure 3.**
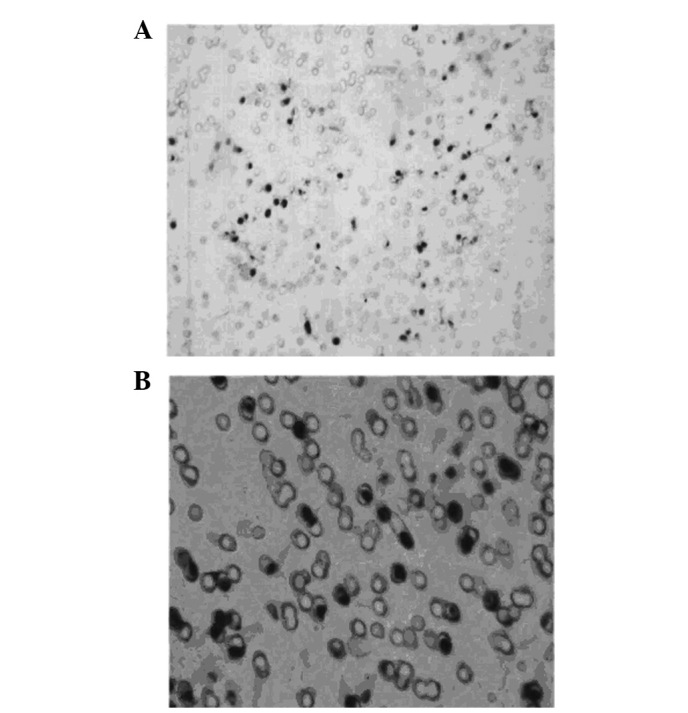
Invasion assay. (A) EJ cells invasion, ×100 magnification; (B) EJ cells invasion, ×200 magnification.

**Figure 4.**
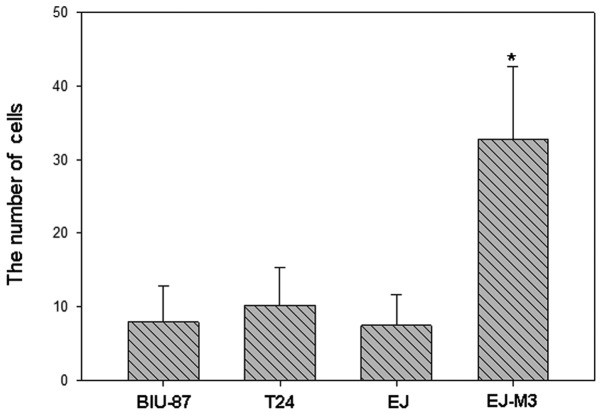
Numbers of EJ-M3, EJ, T24 and BIU-87 cells that invaded into the 24-well plates (^*^P<0.001, vs. BIU-87, T24 and EJ).
